# Entry of Challenge Virus Standard (CVS) -11 into N2a cells via a clathrin-mediated, cholesterol-, dynamin-, pH-dependent endocytic pathway

**DOI:** 10.1186/s12985-019-1186-9

**Published:** 2019-06-13

**Authors:** Jie Gao, Xinyu Wang, Mingxin Zhao, Enhua Liu, Ming Duan, Zhenhong Guan, Yidi Guo, Maolin Zhang

**Affiliations:** Key Laboratory of Zoonosis Research, Ministry of Education, Institute of Zoonosis, College of Veterinary Medicine, Jilin University, 5333 Xian Road, Changchun, 130062 China

**Keywords:** RABV, N2a, Clathrin, Caveolin-1, Endocytosis

## Abstract

**Background:**

Rabies virus (RABV), a member of Lyssavirus of Rhabdoviridae family, is a kind of negative-strand RNA virus. The zoonosis caused by RABV leads to high mortality in animals and humans. Though with the extensive investigation, the mechanisms of RABV entry into cells have not been well characterized.

**Methods:**

Chemical inhibitors and RNA interference (RNAi) were used to analysis RABV internalization pathway. The expression level of viral N protein was examined by quantitative PCR and western blot, and the virus infection in the cells was visualized by fluorescence microscopy.

**Results:**

We firstly examined the endocytosis pathway of the challenge virus standard (CVS) -11 strain in N2a cells. Chlorpromazine treatment and knockdown of clathrin heavy chain (CHC) significantly reduced viral entry, which proved clathrin was required. Meanwhile neither nystatin nor knocking down caveolin-1 (Cav1) in N2a cells had an effect on CVS-11 infection, suggesting that caveolae was independent for CVS-11 internalization. And when cholesterol of cell membrane was extracted by MβCD, viral infection was strongly impacted. Additionally by using the specific inhibitor dynasore and ammonium chloride, we verified that dynamin and a low-pH environment were crucial for RABV infection, which was confirmed by confocal microscopy.

**Conclusions:**

Our results demonstrated that CVS-11 entered N2a cells through a clathrin-mediated, cholesterol-, pH-, dynamin-required, and caveolae-independent endocytic pathway.

**Electronic supplementary material:**

The online version of this article (10.1186/s12985-019-1186-9) contains supplementary material, which is available to authorized users.

## Background

Rabies virus (RABV), a member of the genus *Lyssavirus* within the family *Rhabdoviridae*, which is a neurotropic pathogen, causes encephalomyelitis and high mortality in animals and humans. The virion is a bullet-shaped cylinder range from 100 to 430 nm in length and 45 to 100 nm in diameter that made up of five proteins: nucleocapsid (N) protein, large (L) protein, phosphoprotein (P), glycoprotein (G) and matrix (M) protein. Among them, N, P, L proteins form the ribonucleoprotein complex (RNP) along with the viral RNA genome, which is surrounded by G and M protein [1]. N protein binds to genomic RNA tightly protecting RNA from degradation. M protein beneath the envelop associates with both envelop and RNP, and contributes to viral assembly [2]. The neurotropism of RABV is decided by trans-membrane protein G because G is capable of recognizing receptors that exist in cell surface and plays an important role in fusion events between virus and vesicle membranes [3].

Attachment to membrane mediated by recognition of receptors initiates the infection process, before internalization follows. Till now, multiple receptors like nicotinic acetylcholine receptor (nAChR), neural cell adhesion molecule (NCAM), p75 neurotrophin receptor (p75NTR), and metabotropic glutamate receptor subtype 2 (mGluR2) are identified to be utilized by RABV [4, 5]. The endocytic pathways that viruses enter the host cells include clathrin-mediated, caveolae-mediated, macropinocytosis/phagocytosis and other mechanisms [6, 7]. Most members of Rhabdoviridae family such as Vesicular stomatitis virus (VSV) [8], Australian bat lyssavirus (ABLV) [9], infectious hematopoietic necrosis virus (IHNV) [10] or Bovine ephemeral fever virus (BEFV) internalize host cells through clathrin-mediated endocytosis [11]. Although the study of rabies virus endocytic pathway is not in-depth, several researches on the precise mechanisms of RABV uptake into different kinds of cells have been conducted. Using a recombinant VSV of which the endogenous glycoprotein was replaced with that of RABV (rVSV RABV G), previous studies have found that RABV internalized African green monkey kidney cell line (BS-C-1) [12] and peripheral neurons [13] through clathrin-mediated endocytic pathway by pharmacological perturbations or protein abundance, while G protein is the key factor to facilitate endocytosis. Additionally virus particles were observed by electron micrographs in coated pits in chicken embryo-related (CER) cells [14] and hippocampal neurons [15]. Nonetheless the mechanisms by which RABV enters cells are not well characterized.

Our goals are to discuss the pathway of RABV internalization in neuronal cells and clarify whether CVS-11 enters N2a cells via clathrin- or non-clathrin-mediated endocytosis. In this study we used chemical inhibitors and RNA interference (RNAi) to examine the roles of clathrin and caveolin-1 in the viral entry process. The results indicated that chlorpromazine and knockdown of clathrin heavy chains (CHC) reduced CVS-11 infection, however, CVS-11 entry was not affected by nystatin or knockdown of caveolin-1. In addition, we defined the involvement of cholesterol, dynamin, low-pH in CVS-11 infection through chemical approaches. Ultimately the results will promote our current recognization of Lyssavirus endocytosis mechanism and provide a novel target of antiviral drug development.

## Methods

### Cells and viruses

Neuro-2a cells (N2a) were grown in Dulbecco’s modified Eagle medium (DMEM; CCS30015.03 MRC) supplemented with 10% fetal bovine serum (FBS; A6806–45 NQBB) and maintained in a humidified incubator at 37 °C and 5% CO2. Baby Hamster Syrian Kidney (BHK) cells were cultured in DMEM with 5% FBS. The challenge virus standard strain (CVS) -11 of rabies virus was stored in our laboratory. Virus was propagated in BHK-21 cells. To generate virus stocks, BHK cells were grown in monolayers of T75 flask at 90% confluence and infected with CVS-11 at a multiplicity of infection (MOI) of 0.8, then harvested after 72 h. Virions were collected through three freeze-thaw cycles and centrifugation. Viral titers were determined by calculating the 50% tissue culture infectious dose (TCID50) on N2a cells using the Karber method.

### Cell viability assay

Potential cytotoxic effects of drugs on N2a cells are evaluated by MTT reagent (M5655, Sigma). Briefly, subconfluent cell cultures grown in 96-well plates were incubated with various concentrations of drugs. After incubation for 48 h at 37 °C, 10 μl of the MTT (5 mg/ml) reagent was added to cells. Then after incubation at 37 °C for another 4 h, supernatant was extracted and DMSO (V900090, Sigma) was added, then absorbance at the wave-length of 490 nm was measured by using a plate reader (Tecan) after 15 min.

### Drug treatments and cell infection

For investigating the entry mechanisms of RABV, we used chlorpromazine (C2481, TCI), MβCD (C4555, Sigma), nystatin (N9150, Sigma), dynasore (D7693, Sigma) and ammonium chloride (A9434, Sigma) to treat cells. N2a cell monolayers were seeded into 6-well plates or 24-well plates and pretreated with drugs as listed before for 1 h at 37 °C. After pretreatment, cells were washed with PBS and incubated with CVS at MOI of 0.1 for 1 h at 37 °C. At 3 h and 24 h postinfection (hpi), the viral RNA level was quantitated by using a reverse transcription-quantitative real-time PCR (RT-qPCR) assay and percentage of infection was observed by fluorescence microscopy. At 48 h postinfection (hpi), western blot was performed.

### Real-time qPCR analysis

RNA was extracted from cells using Trizol reagent (9109, TaKaRa). First-strand cDNA was synthesized using a PrimeScript™ RT reagent Kit with gDNA Eraser (RR047A, TaKaRa). RT-qPCR was performed on the 7500 real-time PCR system (Applied Biosystems) according to the manufacturer’s instructions (Invitrogen; Life Technologies Corp, Carlsbad CA, USA) using SYBR green real-time PCR Master Mix (4,913,914,001, Roche). The cycling conditions were as follow: 40 cycles for 95 °C 10 min, 95 °C 15 s, and 60 °C 1 min. The RT-PCR primer sequences are as follow: virus nucleoprotein genome forward primer 5′-GGTTATTGCTCGATGTGCTCCT-3′ and reverse primer 5′-GCCGCCTCGTATTCTTGAAGTT-3′; CHC forward primer 5′-GAACAGAATCAGCGGAGAA-3′ and reverse primer 5′-TCAGAGCCAAGTCAGGAT-3′; caveolin-1 forward primer 5′-AAGGAGAAGATGGAGAAGGAC-3′ and reverse primer 5′-CTTGACGTGGAAGGTGAA-3′; GAPDH forward primer 5′-AGGTCGGTGTGAACGGATTTG-3′ and reverse primer 5′-TGTAGACCATGTAGTTGAGGTCA-3′.

### siRNA transfection

For small interfering RNA (siRNA) analysis, the siCHC for the clathrin heavy chain (CHC) (GGGCCUGCUGCAGCGUGCAUUAGAA) and siCav1 for caveolin-1 (UCCAUACCUUCUGCGAUCCACUCUU) were synthesized by Invitrogen. Stocks (20 μM) were prepared of each siRNA, which were aliquotted and stored at − 20 °C. N2a cells were seeded at 4 × 10^5^ cells/well in 6 well plates and incubated at 37 °C. After adhered to the plastic, the cells were transfected with 25 pmol siRNA. Normal control-siRNA was setup for comparison with the results from the experimental group. The transfection reagents Lipofectamine RNAiMAX (13,778,150, Invitrogen) was used according to the manufacturers’ instructions. After 24 h incubation at 37 °C, the N2a cells were infected with CVS-11 at MOI of 0.1. Cells were harvested and analyzed by qPCR at 3 h and 24 h p.i., western blot at 48 h p.i..

### Western blot

N2a cells were washed with PBS and lysed in a modified radioimmunoprecipitation assay (RIPA) lysis buffer (#9806, Cell Signaling Technology) with 1 mM phenylmethylsulfonyl fluoride (PMSF). Protein concentrations were determined with a BCA Protein Assay kit (#23227, Thermo). An equal amount of protein lysate was separated by 8% or 10% SDS-polyacrylamide gels and transferred to PVDF membranes (3,010,040,001, Roche). Membranes were blocked in TBST containing 5% non-fat dried milk and incubated with primary antibodies overnight at 4 °C. The membranes were washed with TBST and incubated with secondary antibody (1:2000 dilutions in 5% non-fat dried milk) for 2 h at room temperature (RT). Bound antibodies were visualized by chemiluminescent HRP substrate (#32209, Thermo). The mean densities of protein bands were measured by Image J software. The primary antibodies used are as follows: anti-rabies Virus (5B12) (NB110–7542, Novus) (1:1000), GAPDH (1A6) mAb (MB001, Bioworld) (1:5000), Clathrin Heavy Chain (P1663) Antibody (#2410, Cell Signaling Technology) (1:500), Caveolin-1 Antibody (#3238, Cell Signaling Technology) (1:500).

### Immunofluorescence analysis

N2a cells were washed with PBS in the 24-well plate. The cells were fixed with 4% paraformaldehyde for 30 min and permeabilized with 0.1% Triton X-100 for 10 min. After blocked with PBS 0.1% with 5% goat serum for 2 h, the cells were incubated with fluorescein isothiocyanate (FITC) -anti-Rabies Monoclonal antibody (1:200) (800–092, FUJIREBIO) and Evans Blue (1:200) (E2129, Sigma) for 2 h at 37 °C. Fluorescence images were acquired using Olympus confocal (Olympus FV1000 confocal laser scanning microscope, Japan). Images were analyzed using Olympus, Image J and Photoshop software.

### Statistical analysis

All data were presented as the mean standard deviations (SD). Student’s *t*-test was used to evaluate the statistical significance of pairs of treated or untreated groups. *P* < 0.05 represented a statistically significant difference. All statistical analyses and calculations were performed by using GraphPad Prism 5.

## Results

### RABV entry is dependent on clathrin-mediated endocytic pathways

**Fig. 1:**
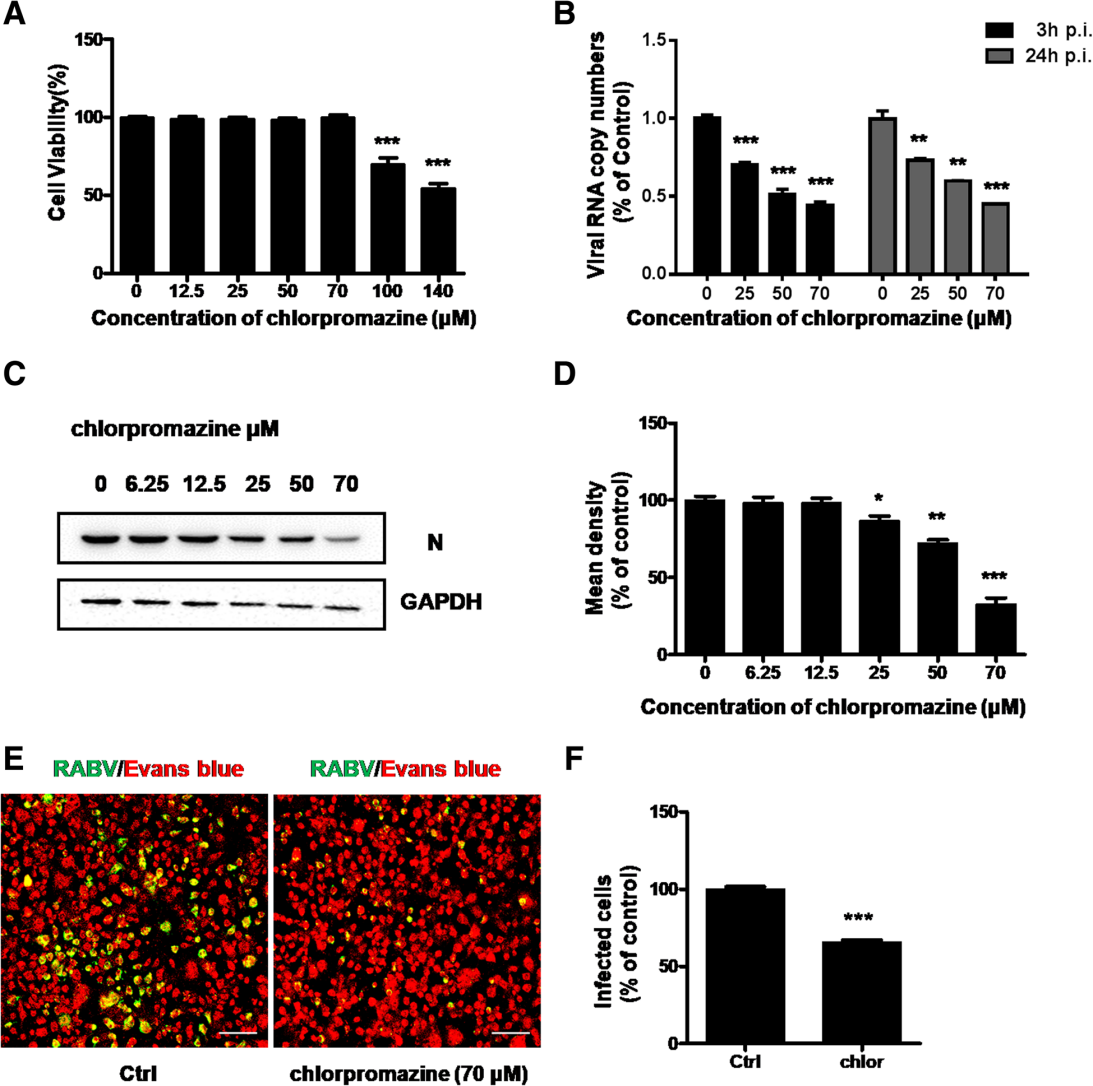
Effect of chlorpromazine on CVS-11 infection on N2a cells. **a** Quantification of cytotoxic effects of chlorpromazine on N2a cells ranging from 0 to 140 μM was examined by MTT assay. **b**, **c** N2a cells were pretreated with increasing concentrations (0 μM, 25 μM, 50 μM, 70 μM) of chlorpromazine for 1 h at 37 °C and infected with CVS-11 (MOI 0.1). At 3 h and 24 h p.i., infected cells were lysed to determine RABV N RNA copy numbers by RT-qPCR (**b**). The cells were lysed and processed for western blot analysis of protein N at 48 h p.i.. GAPDH was used as a loading control (**c**). **d** Relative protein levels were analyzed by using ImageJ. The results are presented as the mean ± SD of three independent experiments. **e** N2a cells were treated with 70 μM chlorpromazine for 1 h and infected with CVS-11 (MOI 0.1). At 24 h p.i., cells were fixed and stained with an FITC-anti-Rabies Monoclonal antibody. Cytoplasm was stained with Evans Blue. Scale bars, 70 μm. **f** The number of infected cells was counted and percentage of infected cells after drug treated compared to control group was assessed. Means and S.D. values are shown. Statistical significances of the differences are indicated. Five fields of about 200 cells were counted. Student’s *t* test, *p* < 0.05 (*); *p* < 0.01 (**); *p* < 0.001 (***)

Previous studies have shown that RABV endocytosis is dependent on clathrin. Therefore we first performed MTT assay to exclude cytotoxic side effects upon chlorpromazine treatment from 0 to 140 μM for 24 h. As shown in Fig. 1a, the viability of N2a cells remained unchanged until up to 100 μM. To test the effect of chlorpromazine on the infection of CVS, N2A cells were pretreated with increasing concentration of drug (0, 25, 50, 70 μM), followed by inoculation with CVS-11 at MOI 0.1 at 37 °C, mRNA was harvested after 3 h and 24 h respectively while the whole cell protein were harvested after 48 h. Viral RNA copy numbers of CVS-11 were both reduced significantly in a dose-dependent manner at 3 h and 24 h p.i. (Fig. 1b), western blot showed the same phenomenon (Fig. 1c, d). According to fluorescence results, CVS-11 infection was effectively reduced to nearly 70% compared with untreated cells (Fig. 1e, f).

**Fig. 2:**
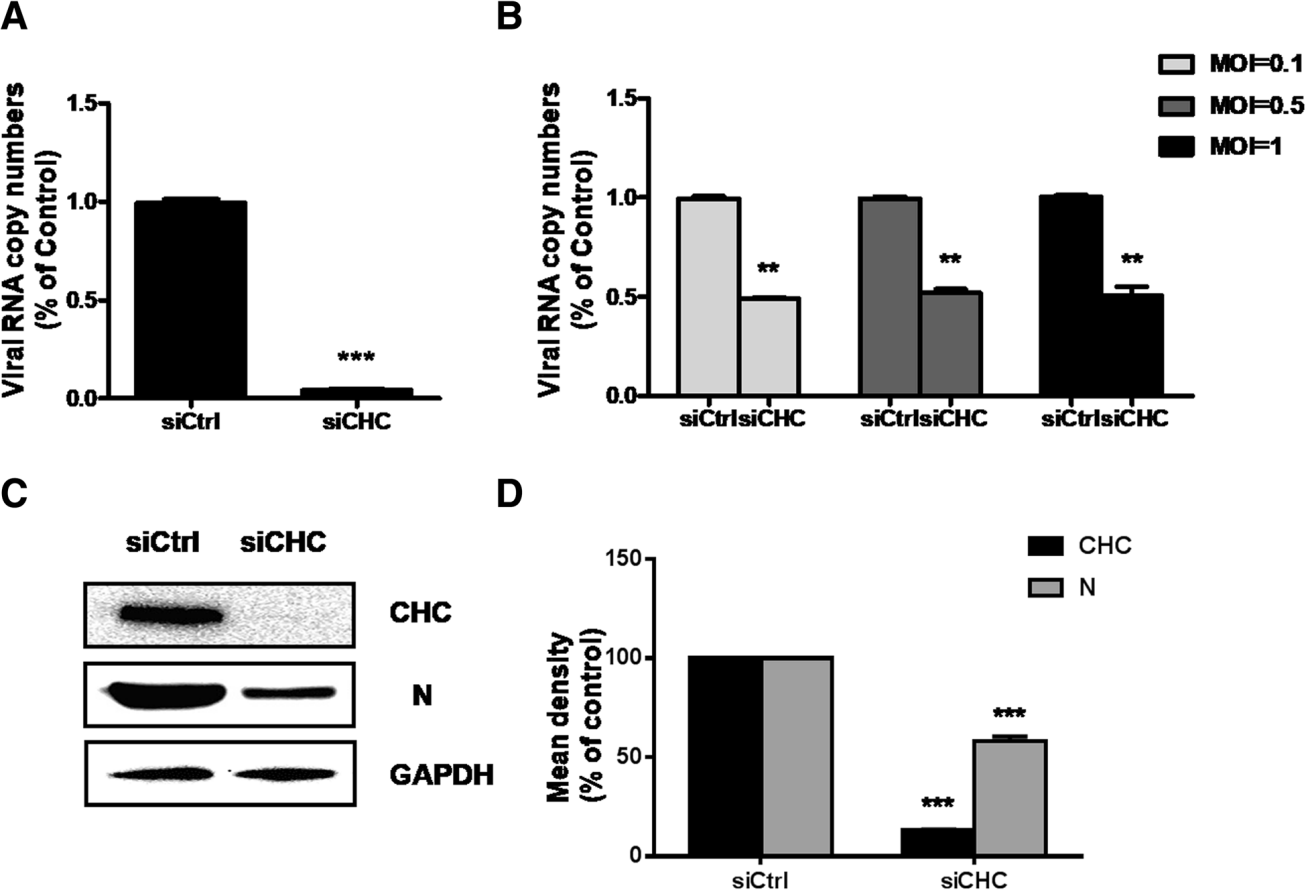
Clathrin-1 is required for CVS-11 entry. **a** CHC knockdown was determined by RT-qPCR. **b** siCtrl- or siCHC- transfected cells were infected with CVS-11 (MOI 0.1, 0.5, 1). At 24 h p.i., the cell was lysed to determine RABV N RNA copy numbers by RT-qPCR. **c**, **d** Results are presented as the means ± SD of data from three independent experiments. Effect of CHC knockdown on CVS-11 infectivity was determined by western blot at 48 h p.i. with anti-RABV, anti-CHC and GAPDH antibodies (**c**). Relative protein levels were analyzed by using ImageJ (**d**). Student’s *t* test, *p* < 0.05 (*); *p* < 0.01 (**); *p* < 0.001 (***)

To confer the role of clathrin during RABV endocytic, we used two independent specific siRNA against heavy chain of clathrin (CHC) and infection with CVS-11. Figure 2a showed that clathrin mRNA level declined to about 5% in transfected N2A cells compared with control siRNA-transfected cells, while mRNA of viral N protein reduced to around 50% when inoculated at MOI 0.1, 0.5 and 1. (Fig. 2b). Along with reduction of CHC, the infection of RABV was significantly blocked, which assumed to be related to clathrin-mediated endocytic progress (Fig. 2c and d). It was verified by western blot assay, while clathrin protein level declined to about 15%, the viral infection reduced to 55% (Fig. 2)d. Taken together, CVS-11 entry to N2a cells required clathrin-mediated pathway, consistent with previously reported results in other cell lines.

### Cholesterol is required for RABV infection

**Fig. 3:**
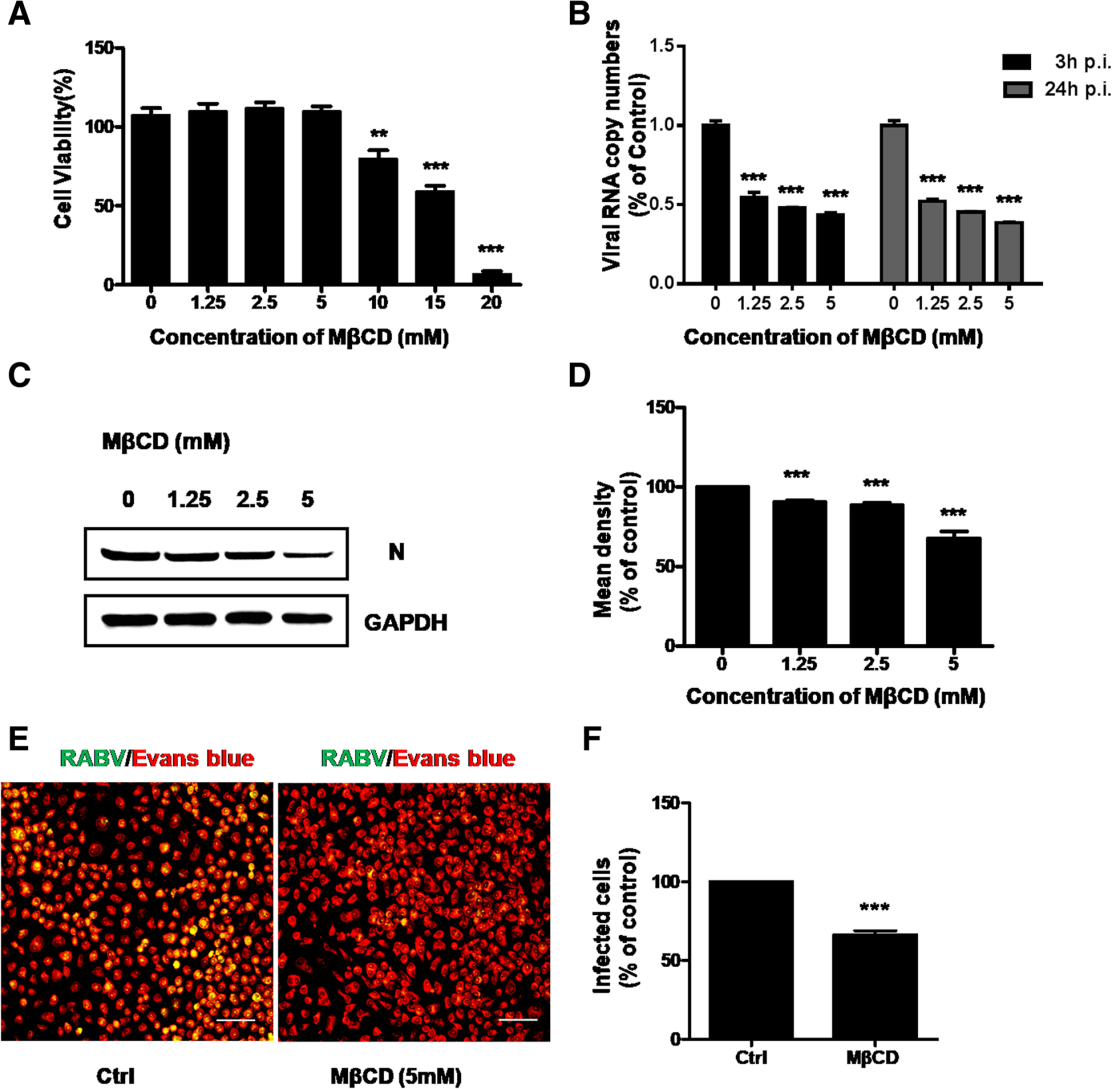
Effect of MβCD on CVS-11 infection in N2a cells. **a** Quantification of cytotoxic effects of MβCD on N2a cells ranging from 0 to 20 mM was examined by MTT assay. **b** N2a cells were pretreated with increasing concentrations (0 mM, 1.25 mM, 2.5 mM, 5 mM) of MβCD for 1 h at 37 °C and infected with CVS-11 (MOI 0.1). At 3 h and 24 h p.i., infected cells were lysed to determine RABV N RNA copy numbers by RT-qPCR. **c** The cells were pretreated with increasing concentrations (0 mM, 1.25 mM, 2.5 mM, 5 mM) of MβCD for 1 h at 37 °C and infected with CVS-11 (MOI 0.1). The cells were lysed and processed for western blot analysis of RABV N protein. GAPDH was used as a loading control. **d** Relative protein levels were analyzed by using ImageJ. The results are presented as the mean ± SD of three independent experiments. **e** N2a cells were treated with 5 mM MβCD for 1 h and infected with CVS-11 (MOI 0.1). At 24 h p.i., cells were fixed and stained with an FITC-anti-Rabies Monoclonal antibody. Cytoplasm was stained with Evans Blue. Scale bars, 70 μm. **f** The number of infected cells was counted and percentage of infected cells after drug treated compared to control group was assessed. Means and S.D. values are shown. Statistical significances of the differences are indicated. Five fields of about 200 cells were counted. Student’s *t* test, *p* < 0.05 (*); *p* < 0.01 (**); *p* < 0.001 (***)

Various viruses enter host cells through lipid rafts in which cholesterol is a predominant component [16–18]. Membrane cholesterol can be selectively extracted by pharmacological agents such as MβCD, resulting in lipid raft disruption. Previous studies reported that deleption of cholesterol effected RABV infection in BHK-21 (RABV susceptible cell line) and HEp-2 (relatively resistant to RABV infection cell line) cells [19]. We conducted a series of experiments to determine if cholesterol is involved in CVS-11 infection in N2a cells. Firstly, N2a cells were treated with 0 to 20 mM MβCD for 24 h to evaluate cytotoxic effects. The cell viability was uneffected until concentration of MβCD increased to 10 mM (Fig. 3a). RT-qPCR assay showed that there was increasingly inhibitory impact on RNA copy numbers of RABV N at 3 h and 24 h p.i. when cells pretreated with 0, 1.25, 2.5, 5 mM MβCD (Fig. 3b). The expression of RABV N showed the similar trend (Fig. 3c and d). The depletion of cholesterol during MβCD treatment was confirmed by BODIPY staining of the cells (Additional file 1: Figure S1). And pretreatment of N2a cells with 5 mM MβCD was verified to inhibit CVS-11 infection compared to that in the untreated control by Immunofluorescence assay (Fig. 3e and f). These data indicated that cholesterol depletion also influenced CVS-11 infection in N2a cells.

### RABV infection is caveolae independent

**Fig. 4:**
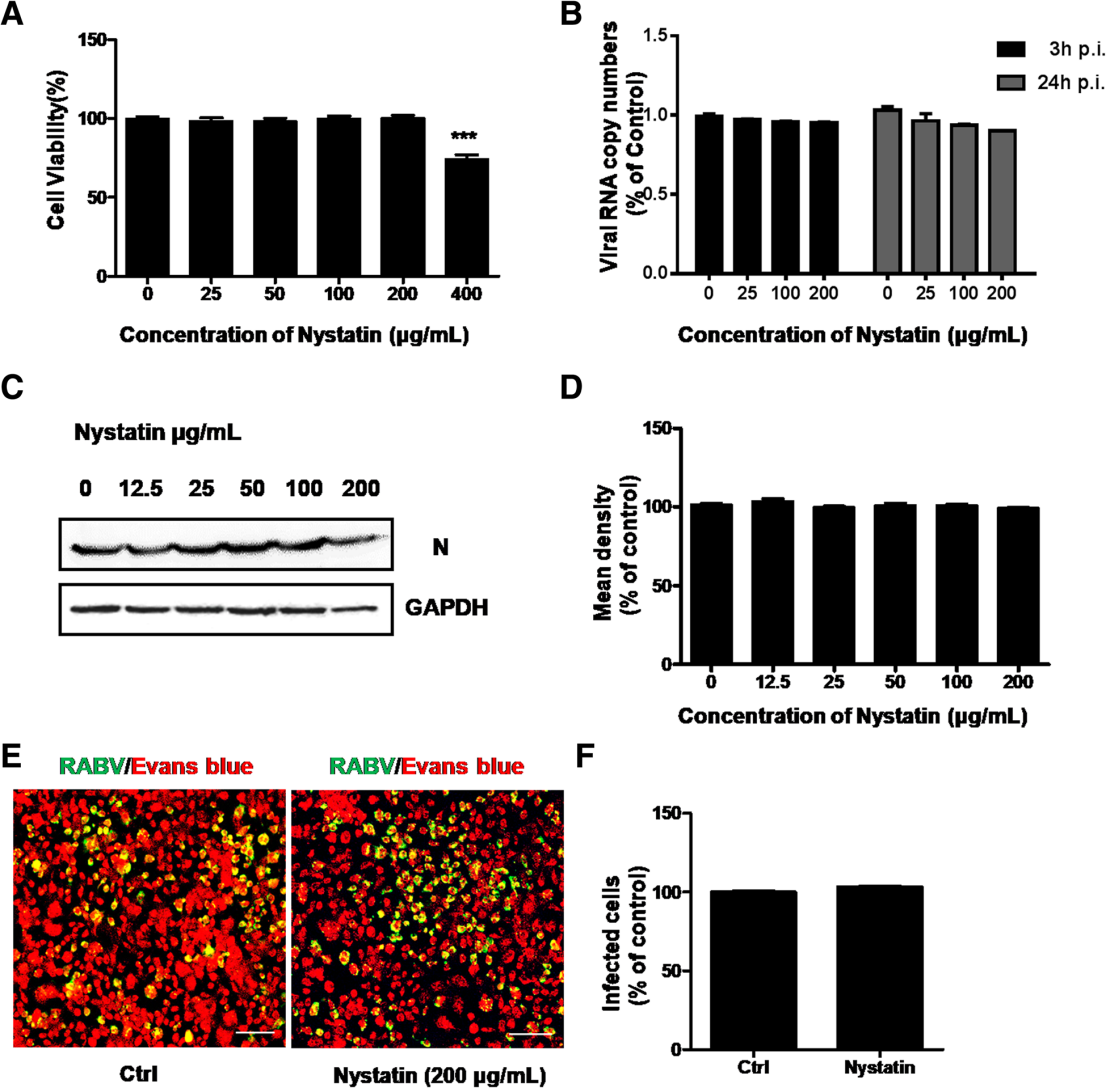
Effect of nystatin on CVS-11 infection in N2a cells. **a** Quantification of cytotoxic effects of nystatin on N2a cells ranging from 0 to 400 μM was examined by MTT assay. **b** N2a cells were pretreated with increasing concentrations (0 μM, 25 μM, 100 μM, 200 μM) of nystatin for 1 h at 37 °C and infected with CVS-11 (MOI 0.1). At 3 h and 24 h p.i., infected cells were lysed to determine RABV N RNA copy numbers by RT-qPCR. **c** The cells were pretreated with increasing concentration (0 μM, 12.5 μM, 25 μM, 50 μM, 100 μM, 200 μM) of nystatin for 1 h at 37 °C and infected with CVS (MOI 0.1). The cells were lysed and processed for western blot analysis of RV-N protein. GAPDH was used as a loading control. **d** Relative protein levels were analyzed by using ImageJ. The results are presented as the mean ± SD of three independent experiments. **e** N2a cells were treated with 200 μM nystatin for 1 h and infected with CVS-11 (MOI 0.1). At 24 h p.i., cells were fixed and stained with an FITC-anti-Rabies Monoclonal antibody. Cytoplasm was stained with Evans Blue. Scale bars, 70 μm. **f** The number of infected cells was counted and percentage of infected cells after drug treated compared to control group was assessed. Means and S.D. values are shown. Statistical significances of the differences are indicated. Five fields of about 200 cells were counted. Student’s *t* test, *p* < 0.05 (*); *p* < 0.01 (**); *p* < 0.001 (***)

To further assess whether caveolae plays a vital role in CVS-11 infection as well as clathrin, here we used nystatin, which inhibit caveolar/raft-dependent entry to treat N2a cell line. Cells were first treated with 0 to 800 μg/mL nystatin for 24 h to evaluate cytotoxic effects. The increasing doses of drugs have no effect on N2a cell viability until the concentration up to 400 μg/mL (Fig. 4a). Pretreated with 0 to 200 μg/mL nystatin and infected with CVS-11, RT-qPCR and western blot were performed. The mRNA level and protein level of RABV N both remained no decrease (Fig. 4b, c and d). The intensity of fluorescently labeled RABV and the percentage of infected cells had no significant change (Fig. 4e and f).

**Fig. 5:**
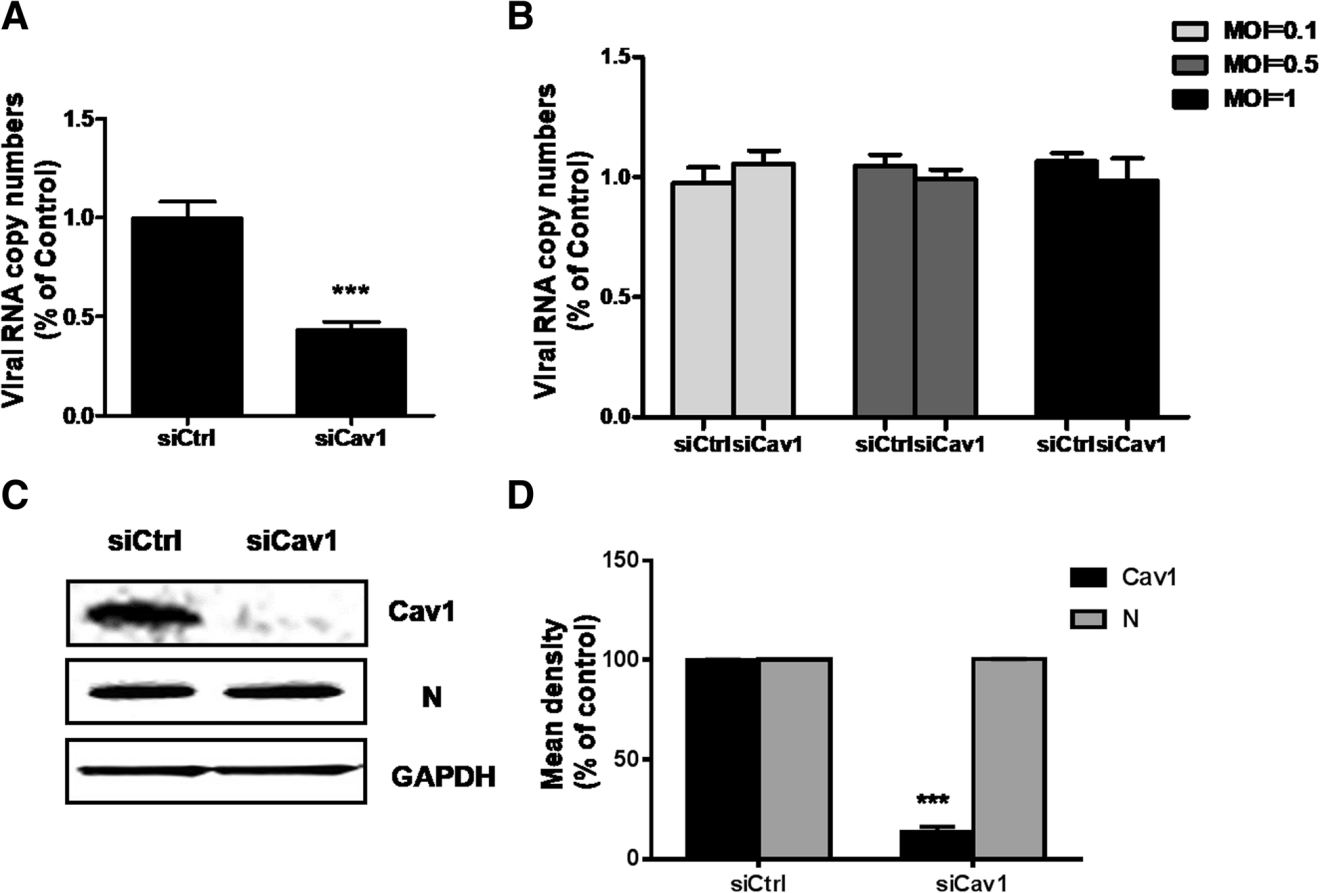
Caveolin-1 is not required for CVS-11 entry. **a** Caveolin-1(Cav1) knockdown was determined by RT-qPCR. **b** siCtrl- or siCav1- transfected cells were infected with CVS-11 (MOI 0.1, 0.5, 1). At 24 h p.i., the cells were lysed to determine RABV N RNA copy numbers by RT-qPCR. **c**, **d** Results are presented as the means ± SD of data from three independent experiments. Effect of Cav1 knockdown on CVS-11 infectivity was determined by western blot at 48 h p.i. with anti-RABV, anti-Cav1 and GAPDH antibodies (**c**). Relative protein levels were analyzed by using ImageJ (**d**). Student’s *t* test, *p* < 0.05 (*); *p* < 0.01 (**); *p* < 0.001 (***)

To confer the role of caveolae during RABV endocytic, we performed caveolin-1(Cav1) knockdown to assess the infection of RABV. Cav-1-KD caused caveolin-1 expression decreased, showed in Fig. 5a. Next, N2a cells were transfected with si-Ctrl and si-Cav1 and then infected with CVS-11 at MOI of 0.1, 0.5 and 1. As expected, RABV N RNA level was not reduced in si-Cav-1 transfected cells compared to control group (Fig. 5b). We examined the presence of infected cells by western blot, and found that Cav-1-KD resulted in no inhibition of the number of CVS-11-infected cells (Fig. 5c and d). Above all these results we concluded that CVS-11 infection in N2a cells was undependent on caveolae.

### RABV entry is dependent on dynamin

**Fig. 6:**
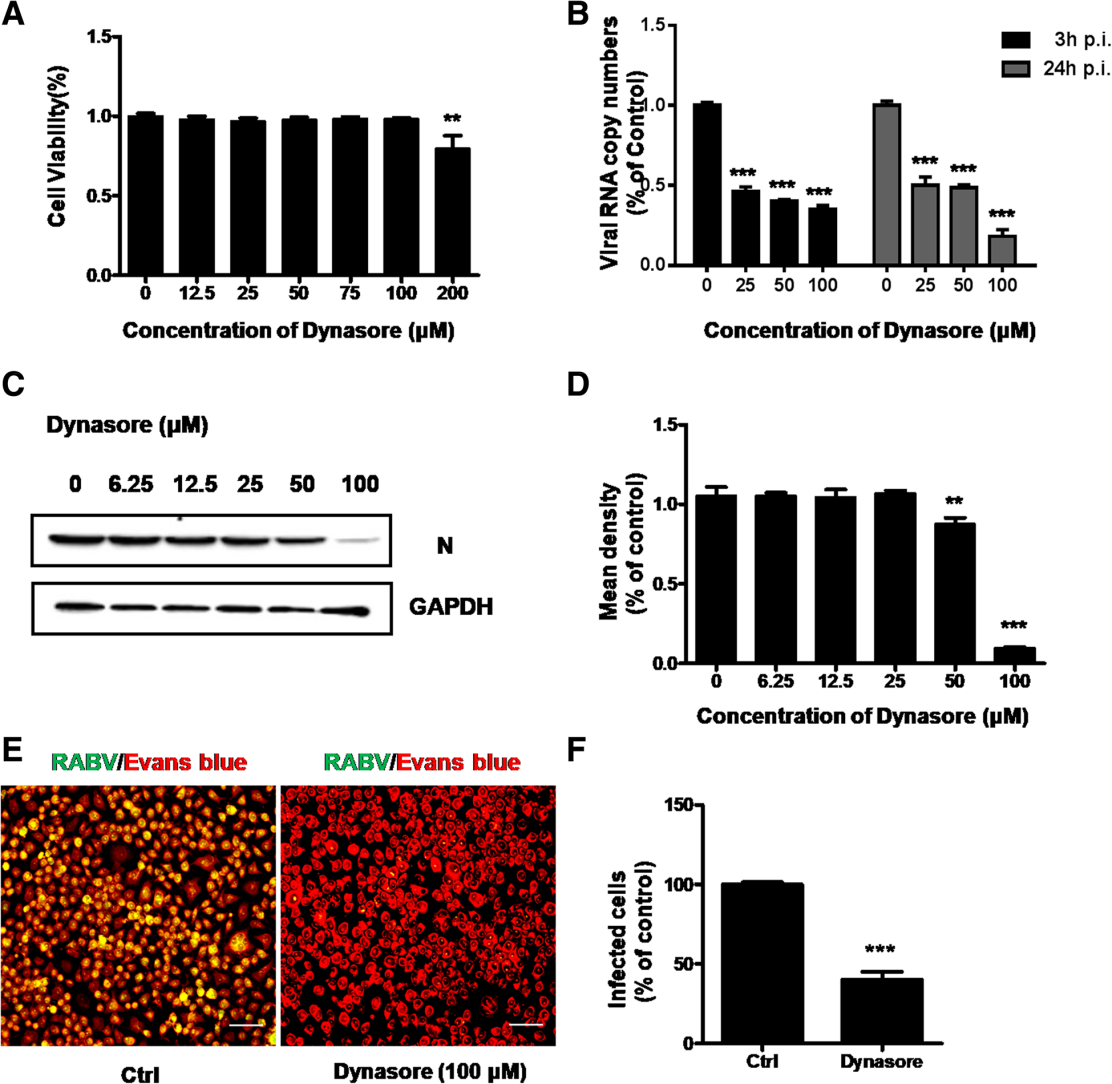
Effect of dynasore on CVS-11 infection in N2a cells. **a** Quantification of cytotoxic effects of dynasore on N2a cells ranging from 0 to 200 μM was examined by MTT assay. **b** N2a cells were pretreated with increasing concentrations (0 μM, 25 μM, 50 μM, 100 μM) of dynasore for 1 h at 37 °C and infected with CVS-11 (MOI 0.1). At 3 h and 24 h p.i., infected cells were lysed to determine RABV N RNA copy numbers by RT-qPCR. **c** The cells were pretreated with increasing concentration (0 μM, 6.25 μM, 12.5 μM, 25 μM, 50 μM, 100 μM) of dynasore for 1 h at 37 °C and infected with CVS-11 (MOI 0.1). The cells were lysed and processed for western blot analysis of RABV N protein. GAPDH was used as a loading control. **d** Relative protein levels were analyzed by using ImageJ. The results are presented as the mean ± SD of three independent experiments. **e** N2a cells were treated with 100 μM dynasore for 1 h and infected with CVS-11 (MOI 0.1). At 24 h p.i., cells were fixed and stained with an FITC-anti-Rabies Monoclonal antibody. Cytoplasm was stained with Evans Blue. Scale bars, 70 μm. **f** The number of infected cells was counted and percentage of infected cells after drug treated compared to control group was assessed. Five fields of about 200 cells were counted. Means and S.D. values are shown. Statistical significances of the differences are indicated. Student’s *t* test, *p* < 0.05 (*); *p* < 0.01 (**); *p* < 0.001 (***)

Dynamin is both involved in clathrin-mediated and caveolar/raft-mediated internalization, and is responsible for scission of plasma membrane through location around the neck of the endocytic indentations. To further determine whether dynamin is required in RABV entry into N2a cell line, dynasore, a dynamin GTPase activity inhibitor, was employed on N2a cells. Cytotoxic effects on N2a cells of dynasore were measured after drug treatment for 24 h. Figure 6a shows that cellular cytotoxicity remained unchanged until the concentration up to 200 μM. The inhibition on RABV infection was significant according to RT-qPCR results (Fig. 6b) and was almost complete when the concentration tolerated to 100 μM in western blot data (Fig. 6c and d). Reduction of fluorescently labeled RABV was observed upon dynasore pretreatment 60% fewer CVS-11-infected cells were observed (Fig. 6e and f). These results above all supported that RABV endocytic depends on dynamin scission.

### RABV entry requires a low pH condition

**Fig. 7:**
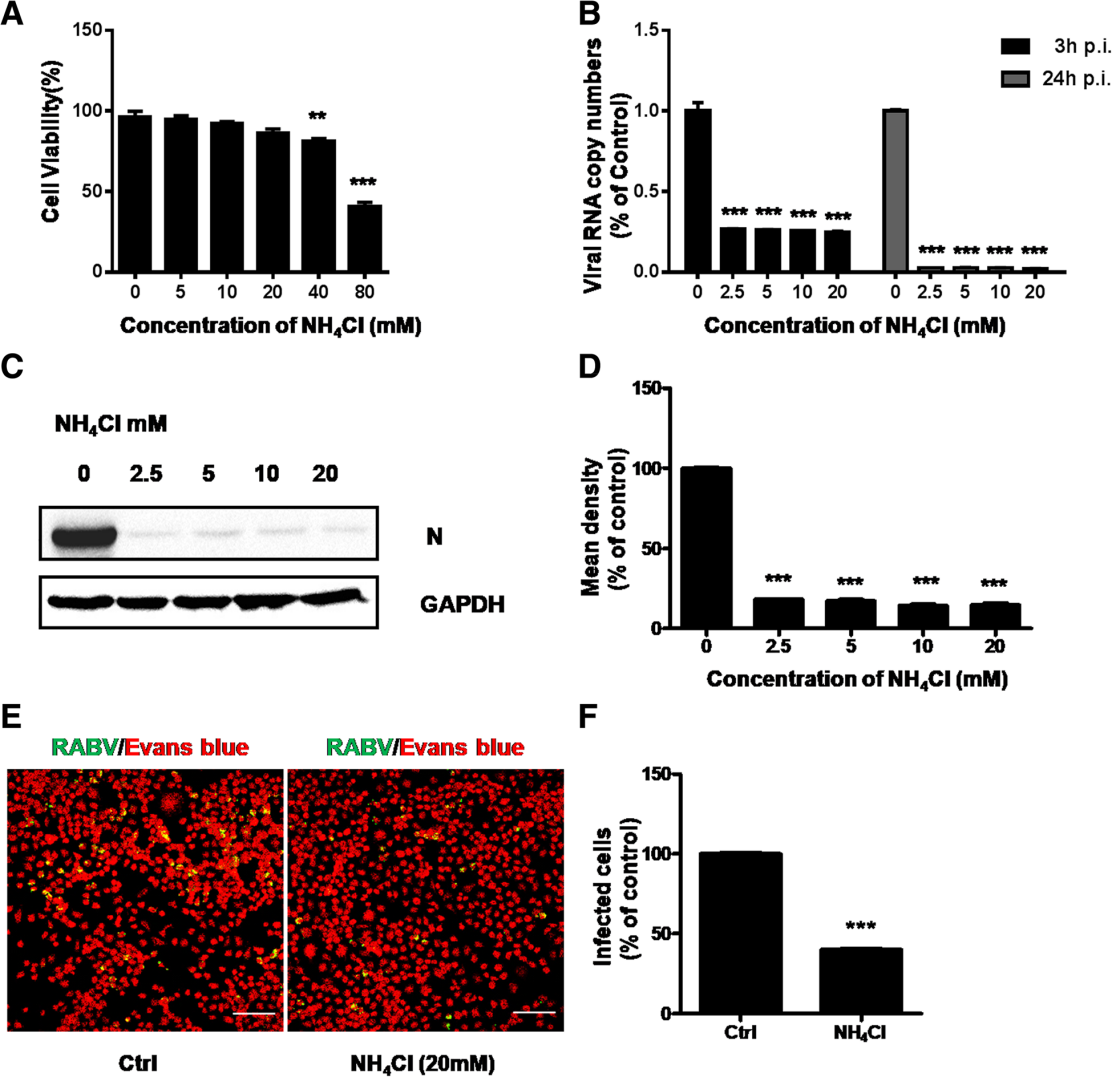
Effect of NH_4_Cl on CVS-11 infection in N2a cells. **a** Quantification of cytotoxic effects of NH_4_Cl on N2a cells ranging from 0 to 80 mM was examined by MTT assay. **b** N2a cells were pretreated with increasing concentrations (0 mM, 2.5 mM, 5 mM, 10 mM, 20 mM) of NH_4_Cl for 1 h at 37 °C and infected with CVS-11 (MOI 0.1). At 3 h and 24 h p.i., infected cells were lysed to determine RABV N RNA copy numbers by RT-qPCR. **c** The cells were pretreated with increasing concentration (0 mM, 2.5 mM, 5 mM, 10 mM, 20 mM) of NH_4_Cl for 1 h at 37 °C and infected with CVS-11 (MOI 0.1). The cells were lysed and processed for western blot analysis of RABV N protein. GAPDH was used as a loading control. **d** Relative protein levels were analyzed by using ImageJ. The results are presented as the mean ± SD of three independent experiments. **e** N2a cells were treated with 20 mM NH_4_Cl for 1 h and infected with CVS-11 (MOI 0.1). At 24 h p.i., cells were fixed and stained with an FITC-anti-Rabies Monoclonal antibody. Cytoplasm was stained with Evans Blue. Scale bars, 70 μm. **f** The number of infected cells was counted and percentage of infected cells after drug treated compared to control group was assessed. Five fields of about 200 cells were counted. Means and S.D. values are shown. Statistical significances of the differences are indicated. Student’s *t* test, *p* < 0.05 (*); *p* < 0.01 (**); *p* < 0.001 (***)

When cells are treated with weak bases such as ammonium chloride (NH_4_Cl), the pHs of intracellular environment are elevated and processes in the endosomal/lysosomal system are inhibited. A low pH condition is crucial for viral entry and intracellular trafficking according to previous reports, so we studied whether it also affected CVS-11 infection in N2a cells in the presence of NH_4_Cl. NH_4_Cl had no effect on N2a viability until the concentration of 20 mM (Fig. 7a). The expression level of RABV N (Fig. 7c and d) as well as the RNA level (Fig. 7b) was significantly inhibited in a dose-dependent manner and nearly abolished even at the minimum concentration (2.5 mM), when treated with NH_4_Cl ranging from 0 to 20 mM prior to infection with CVS-11. The number of infected cells was significantly reduced after pretreatment with 20 mM NH_4_Cl by fluorescence analysis (Fig. 7e and f). All above demonstrated that NH_4_Cl treatment supressed CVS-11 infection in N2a cells. Bafilomycin A1 (Baf-A1) can also influence the intracellular low pH condition via specifically inhibiting vacuolar-type proton (V-H+) pump [20–22]. We also obtained the similar results when cells were treated with Baf-A1 (Additional file 2: Figure S2). Consequently RABV entry requires a low pH condition.

## Discussion

Though rabies virus has been studied for hundreds of years, the researches about the mechanisms of the entry into infectious cell still remain few. In this study, we investigated for the first time the N2a cells entry process of CVS-11 particles through a clathrin-mediated, cholesterol-, dynamin-, pH-dependent endocytic pathway.

Viruses utilize different endocytic pathways to enter host cells. Among these, clathrin-mediated endocytosis is most frequently used by many viruses. Previous studies used a recombinant VSV of which the endogenous glycoprotein was replaced with that of RABV and found that clathrin-mediated pathway was utilized in African green monkey kidney cell line (BS-C-1) and peripheral neurons [12, 13]. To further examine the internalization of CVS-11 into N2a cells, we initially used chemical inhibitor (chloroquine) and siRNA targeting the heavy chain of clathrin (CHC) to disrupt the clathrin-dependent entry pathway. As demonstrated previously, virus infection was significantly inhibited, suggesting that CVS-11 entry needed clathrin involved. Cholesterol is a prominent component of lipid rafts and play vital roles in virus entry [23–25]**.** It was reported that the cholesterol depletion leaded to increase in RABV adsorption and infection in both BHK-21 and HEp-2 cells [19]. In the present work, N2a cells were treated with chemical drug of MβCD to deplete cholesterol, but opposite results were shown that membrane cholesterol was an absolute requirement for CVS-11 infection, which was consistent with the cholesterol’s function in formation of clathrin-coated endocytic vesicles [26]. Since membrane cholesterol is also required for caveolae formation [27], we next examined whether caveolae played any role in CVS-11 internalization. To specifically inhibit caveolae-mediated endocytosis, nystatin was added and siRNA was used to knockdown the expression of caveolin-1 (Cav1). CVS-11 infection was not affected, so we concluded that CVS-11 entry into N2a cells was caveolae independent. Dynamin as a GTPase mediates membrane fusion required for clathrin-mediated endocytosis [13]. The essential role of dynamin in CVS-11 entry process was also determined from dynasore markedly decreasing CVS-11 infection in N2a cells. The low-pH dependence of CVS-11 infection could easily be speculated from the reduction of viral infection after ammonium chloride treatment.

## Conclusions

In this study, we used chemical inhibitors and siRNA to dissect the internalization mechanism of CVS-11 in N2a cells for the first time. Evidences presented here demonstrated that CVS-11 entry was mediated by clathrin-, cholesterol-, dynamin- and pH-dependent, but not caveolin-1 dependent, pathway in N2a cells. Our studies have supplemented the deficiency of RABV entry-related researches and contributed to better understanding the RABV pathogenic mechanisms.

## Additional files

Additional file 1: **Figure S1.** MβCD treatment caused lipid disruption in N2a cells. N2a cells, treated (or mock-treated) with 5 mM MβCD for 2 h at 37 °C were fixed and pulse-labeled for 20 min with BODIPY (green). Nuclei were stained with DAPI (blue). Scale bars, 10 μm. (TIF 78 kb)

Additional file 2: **Figure S2.** Effect of Bafilomycin A1 on CVS-11 infection in N2a cells. A Quantification of cytotoxic effects of Bafilomycin A1 on N2a cells ranging from 0 to 80 nM was examined by MTT assay. B N2a cells were pretreated with increasing concentrations (0 nM, 5 nM, 20 nM, 40 nM) of Bafilomycin A1 for 1 h at 37 °C and infected with CVS-11 (MOI 0.1). At 3 h and 24 h p.i., infected cells were lysed to determine RABV N RNA copy numbers by RT-qPCR. C The cells were pretreated with increasing concentration (0 nM, 2.5 nM, 5 nM, 10 nM, 20 nM, 40 nM) of Bafilomycin A1 for 1 h at 37 °C and infected with CVS-11 (MOI 0.1). The cells were lysed and processed for western blot analysis of RABV N protein. GAPDH was used as a loading control. D Relative protein levels were analyzed by using ImageJ. The results are presented as the mean ± SD of three independent experiments. E N2a cells were treated with 40 nM Bafilomycin A1 for 1 h and infected with CVS-11 (MOI 0.1). At 24 h p.i., cells were fixed and stained with an FITC-anti-Rabies Monoclonal antibody. Cytoplasm was stained with Evans Blue. Scale bars, 70 μm. F The number of infected cells was counted and percentage of infected cells after drug treated compared to control group was assessed. Five fields of about 200 cells were counted. Means and S.D. values are shown. Statistical significances of the differences are indicated. Student’s *t* test, *p* < 0.05(*); *p* < 0.01 (**); *p* < 0.001(***). (TIF 637 kb)

## Data Availability

Not applicable.

## Abbreviations

ABLV: Australian bat lyssavirus
Baf-A1: Bafilomycin A1
BEFV: bovine ephemeral fever virus
BHK: Baby hamster kidney cells
Cav1: Caveolin-1
CER: Chicken embryo-related cells
CHC: Clathrin heavy chain
CVS: Challenge virus standard
FBS: Fetal bovine serum
FITC: Fluorescein isothiocyanate
hpi: Hour post infection
IHNV: Infectious hematopoietic necrosis virus
mGluR2: Metabotropic glutamate receptor subtype 2
MOI: Multiplicity of infection
MβCD: Methylated-β-Cyclodextrin
N2a: Neuro-2a cells
nAChR: Nicotinic acetylcholine receptor
NCAM: Neural cell adhesion molecule
p75NTR: p75 neurotrophin receptor
PMSF: Phenylmethylsulfonyl fluoride
RIPA: Radioimmunoprecipitation assay lysis buffer
RNP: Ribonucleoprotein
RT: Room temperature
RT-qPCR: Reverse transcription-quantitative Polymerase Chain Reaction
SD: Standard deviations
siRNA: Small interfering RNA
VSV: Vesicular stomatitis virus
