# Supplementary figures and images for: Entry of Challenge Virus Standard (CVS) -11 into N2a cells via a clathrin-mediated, cholesterol-, dynamin-, pH-dependent endocytic pathway
Source: Virol J. 2019 Jun 13;16:80. doi: 10.1186/s12985-019-1186-9 (PMC6567506; doi:10.1186/s12985-019-1186-9)

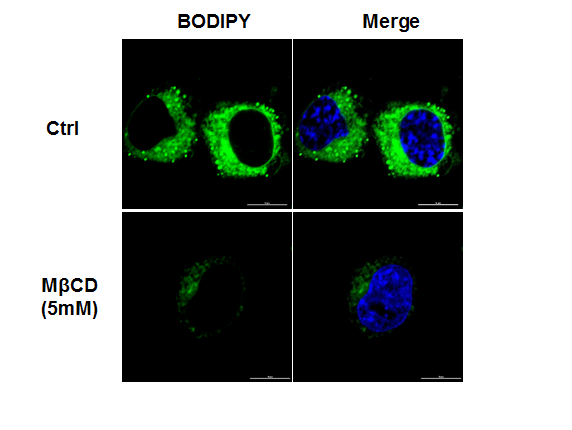

Supplement: Supplementary file 1 — Figure S1. MβCD treatment caused lipid disruption in N2a cells. N2a cells, treated (or mock-treated) with 5 mM MβCD for 2 h at 37 °C were fixed and pulse-labeled for 20 min with BODIPY (green). Nuclei were stained with DAPI (blue). Scale bars, 10 μm. (TIF 78 kb) [file 12985_2019_1186_MOESM1_ESM.tif]

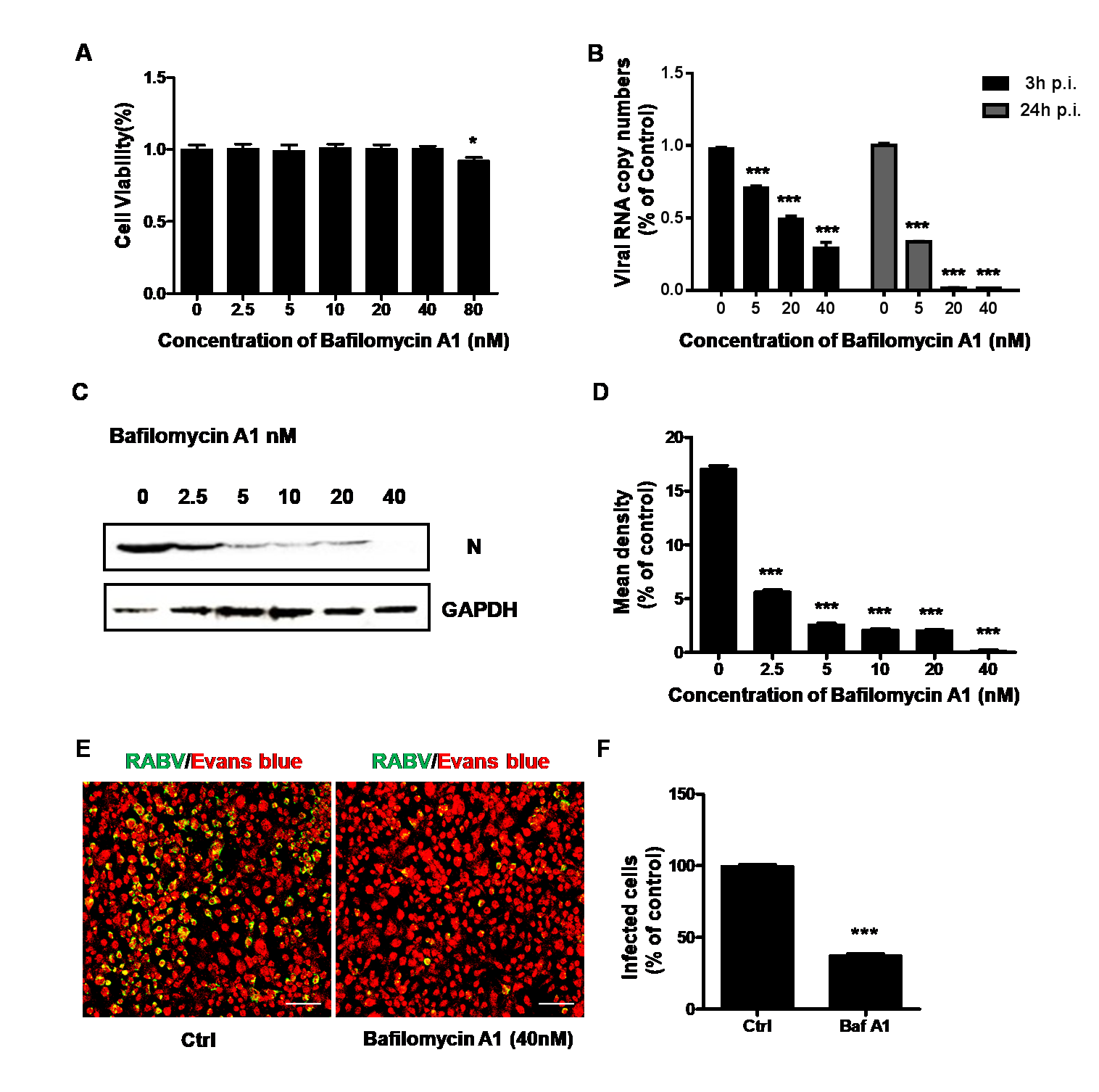

Supplement: Supplementary file 2 — Figure S2. Effect of Bafilomycin A1 on CVS-11 infection in N2a cells. A Quantification of cytotoxic effects of Bafilomycin A1 on N2a cells ranging from 0 to 80 nM was examined by MTT assay. B N2a cells were pretreated with increasing concentrations (0 nM, 5 nM, 20 nM, 40 nM) of Bafilomycin A1 for 1 h at 37 °C and infected with CVS-11 (MOI 0.1). At 3 h and 24 h p.i., infected cells were lysed to determine RABV N RNA copy numbers by RT-qPCR. C The cells were pretreated with increasing concentration (0 nM, 2.5 nM, 5 nM, 10 nM, 20 nM, 40 nM) of Bafilomycin A1 for 1 h at 37 °C and infected with CVS-11 (MOI 0.1). The cells were lysed and processed for western blot analysis of RABV N protein. GAPDH was used as a loading control. D Relative protein levels were analyzed by using ImageJ. The results are presented as the mean ± SD of three independent experiments. E N2a cells were treated with 40 nM Bafilomycin A1 for 1 h and infected with CVS-11 (MOI 0.1). At 24 h p.i., cells were fixed and stained with an FITC-anti-Rabies Monoclonal antibody. Cytoplasm was stained with Evans Blue. Scale bars, 70 μm. F The number of infected cells was counted and percentage of infected cells after drug treated compared to control group was assessed. Five fields of about 200 cells were counted. Means and S.D. values are shown. Statistical significances of the differences are indicated. Student’s t test, p < 0.05(*); p < 0.01 (**); p < 0.001(***). (TIF 637 kb) [file 12985_2019_1186_MOESM2_ESM.tif]
